# Transcriptional and metabolic signatures of Arabidopsis responses to chewing damage by an insect herbivore and bacterial infection and the consequences of their interaction

**DOI:** 10.3389/fpls.2014.00441

**Published:** 2014-09-17

**Authors:** Heidi M. Appel, Shahina B. Maqbool, Surabhi Raina, Guru Jagadeeswaran, Biswa R. Acharya, John C. Hanley, Kathryn P. Miller, Leonard Hearnes, A. Daniel Jones, Ramesh Raina, Jack C. Schultz

**Affiliations:** ^1^Plant Sciences, Bond Life Sciences Center, University of MissouriColumbia, MO, USA; ^2^Department of Biology, Syracuse UniversitySyracuse, NY, USA; ^3^Department of Genetics, Albert Einstein College of MedicineBronx, NY, USA; ^4^Department of Biochemistry and Molecular Biology, Oklahoma State University - StillwaterStillwater, OK, USA; ^5^Department of Biology, Pennsylvania State UniversityUniversity Park, State College, PA, USA; ^6^Department of Chemistry, Pennsylvania State UniversityUniversity Park, State College, PA, USA; ^7^Allergan, Inc.Irvine, CA, USA; ^8^Department of Pediatrics, Nemours/AI duPont Hospital for ChildrenWilmington, DE, USA; ^9^Department of Statistics, University of MissouriColumbia, MO, USA; ^10^Departments of Biochemistry and Molecular Biology and Chemistry, Michigan State UniversityEast Lansing, MI, USA

**Keywords:** *Arabidopsis thaliana*, *Spodoptera exigua*, *Pseudomonas syringae*, herbivory, hormone signaling, glucosinolates

## Abstract

Plants use multiple interacting signaling systems to identify and respond to biotic stresses. Although it is often assumed that there is specificity in signaling responses to specific pests, this is rarely examined outside of the gene-for-gene relationships of plant-pathogen interactions. In this study, we first compared early events in gene expression and later events in metabolite profiles of *Arabidopsis thaliana* following attack by either the caterpillar *Spodoptera exigua* or avirulent (DC3000 *avrRpm1*) *Pseudomonas syringae* pv. tomato at three time points. Transcriptional responses of the plant to caterpillar feeding were rapid, occurring within 1 h of feeding, and then decreased at 6 and 24 h. In contrast, plant response to the pathogen was undetectable at 1 h but grew larger and more significant at 6 and 24 h. There was a surprisingly large amount of overlap in jasmonate and salicylate signaling in responses to the insect and pathogen, including levels of gene expression and individual hormones. The caterpillar and pathogen treatments induced different patterns of expression of glucosinolate biosynthesis genes and levels of glucosinolates. This suggests that when specific responses develop, their regulation is complex and best understood by characterizing expression of many genes and metabolites. We then examined the effect of feeding by the caterpillar *Spodoptera exigua* on Arabidopsis susceptibility to virulent (DC3000) and avirulent (DC3000 *avrRpm1*) *P. syringae* pv. tomato, and found that caterpillar feeding enhanced Arabidopsis resistance to the avirulent pathogen and lowered resistance to the virulent strain. We conclude that efforts to improve plant resistance to bacterial pathogens are likely to influence resistance to insects and vice versa. Studies explicitly comparing plant responses to multiple stresses, including the role of elicitors at early time points, are critical to understanding how plants organize responses in natural settings.

## Introduction

In the wild, plants experience insect and pathogen attacks at the same time or in close succession and must detect and respond to them in a coordinated way. Responses to one may influence responses to another, and antagonistic, neutral, and synergist effects of plant microbial infection on insect performance have been reported (reviewed in Stout et al., 2006; Barrett and Heil, 2012; Biere and Bennett, 2013; Tack and Dicke, 2013). For example, when Arabidopsis plants are pre-treated with microbes the effect on insect performance varies with the microbial treatment and the herbivore. When plants were treated with microbes to cause systemic acquired resistance (SAR) or induced systemic resistance (ISR), growth of one species of caterpillars was reduced and the other unaffected (Van Oosten et al., 2008). Similarly, when a systemic hypersensitive response (HR) was elicited by avirulent *Pseudomonas syringae*, caterpillar growth was reduced whereas plants treated with the virulent form of that bacterium supported better caterpillar growth (Cui et al., 2002, 2005; Groen et al., 2013). In Arabidopsis, the reverse effect of herbivore feeding on subsequent pathogen attack is even less well studied. Plants pre-treated with caterpillar herbivory were more resistant to bacterial and viral pathogens, including *P. syringae* (De Vos et al., 2006). As a result, we now know that attack by insects or pathogens can affect plant response to the other, but we have little understanding of how or when these interactions occur.

The interaction of plant responses to multiple stresses is assumed to arise from crosstalk in the major signaling pathways. Plant responses to insects and necrotrophic pathogens are thought to be mediated primarily by the jasmonate (JA) and/or ethylene pathways, whereas plant responses to biotrophic pathogens are mediated primarily by the salicylic acid (SA) pathway. However, there is significant crosstalk between them and modulation from other hormones, especially ethylene and abscisic acid (reviewed in Pieterse et al., 2012). The crosstalk hypothesis is partially supported by work with signaling mutants, but it is best evaluated by experiments in which plant gene expression is measured at early time points after insect and/or pathogen attack when the specificity of response is likely to be observed. In this study, we first examined early events in gene expression and later events in metabolite profiles of Arabidopsis following attack by either the caterpillar or the avirulent *P. syringae* to determine the degree of overlap in plant response. We then examined the effect of feeding by the caterpillar *Spodoptera exigua* on Arabidopsis response to virulent and avirulent *P. syringae* pv. *tomato*.

## Materials and methods

### Plant rearing

*Arabidopsis thaliana* (ecotype Columbia) were planted in MetroMix 200 and grown in a growth chamber at 22°C, 66% humidity, 8:16 L:D; and 80 μE illumination. The plants were watered every 2–4 days as needed and fertilized every 2 weeks (Miracle Gro 21-7-7). The plants were used in experiments 6 weeks after germination; at this time, their rosette diameter exceeded the 2 inch pots but they had not yet started bolting.

### Caterpillar rearing

*Spodoptera exigua* (Hübner) were reared by Benzon Research on artificial diet at 29°C and shipped to us as first instar larvae. They developed on artificial diet at 25°C until late second instar larvae. The day before experiments they were acclimated to *Arabidopsis*. The morning of the experiment they were early third instar larvae and were transferred to experimental plants.

### Pathogen preparation

*Pseudomonas syringae* pv. tomato (DC3000vir and DC3000 *avrRpm1*) were cultured overnight in King's Broth, pelleted at 6000 rpm, washed 3X with 10 mM MgSO_4_, then diluted to 5 × 10^7^ cfu in 10 mM MgSO_4_. The pathogen was introduced into six leaves of each plant by syringe, with 10 mM MgSO_4_ as the inoculation control.

### RNA isolation for microarray analysis

Leaf tissue was ground in liquid N by mortar and pestle and RNA isolated by the TRIzol method (Invitrogen) with a sodium acetate final wash. RNA was treated for DNase using TURBO DNase kit (Ambion), and cleaned with RNeasy columns (Qiagen).

### Preparation of cDNA clones

*A. thaliana* cDNA clones were isolated from 10 cDNA libraries constructed by SSH as described (Mahalingam et al., 2003). Also included *A. thaliana* full-length cDNA plasmid clones of corresponding expressed sequence tags (ESTs) generated from the Arabidopsis Biological Resource Center (Columbus, OH) and various other miscellaneous clones. The inserts of cDNA clones were amplified from fresh overnight grown bacterial cultures in 96-well plates as a 100 μl reaction by PCR using primers that were complementary to vector sequences flanking both sides of the cDNA insert. PCR products were purified using QIAquick-96 columns (Qiagen, Valencia, CA) and analyzed by electrophoresis on 1% agarose gel to confirm amplification quality and quantity. The samples were then lyophilized and resuspended in 10 μl of 3XSSC and transferred to 384-well plates for array printing.

### Preparation of cDNA microarray

Microscopic glass slides (Gold Seal, Portsmouth, NH) were surface coated with 3-aminopropyltriethoxysilane (Sigma) and used for printing microarrays at Syracuse University. PCR amplified DNA samples were arrayed in quadruplets from 384-well plate with spot size of 100 and 190 μm a center-to-center spacing onto silane-coated slides using OmniGrid™ (GeneMachine, San Carlos, CA) as a printing device with 4 stealth micro-spotting pins (SMP3: TeleChem, Sunny-vale, CA). After printing, the arrays were dried and stored inside the desiccator (Nalgene, Rochester, NY) till use. The printed array was tested to assess microarray probe and printing quality by staining one or two slides with Syto-61 (Molecular Probes, Eugene, OR). The resulting arrays (22 × 20 mm) contained ~1100 elements containing 209 *A. thaliana* ESTs, and >800 cDNA clones. As an external/positive control, 10 PCR amplified products, non-homologous to any nucleic acid sequences in GenBank, corresponding to mRNA spikes, were used (0.1 μg/μl of each: Stratagene), poly(dA)_50_ oligonucleotide (0.01 μg/μl: Stratagene) to assess the non-specific hybridization due to cDNA containing a poly T track, Salmon Sperm DNA (0.1 μg/μl: Stratagene), Human β-actin PCR product (0.1 μg/μl: Stratagene), Human Cot-1 DNA (0.1 μg/μl: Stratagene), 3XSSC buffer and blank as negative controls. All control DNA sample were spotted in each block of the array. Blank and 3XSSC spots were printed at several locations of the microarray to assess background and check for carry-over between samples. The array also contains 12 important marker genes such as *PR1, PDF, Actin, HEL* etc. (Supplemental Table 1) as internal controls to assess the effectiveness of each treatment.

### Fluorescent probe preparation and microarray hybridization

For microarray hybridizations, total RNA was used to synthesize fluorescence-labeled probes. Briefly, 35 μg of total RNA was reverse transcribed by using Power script reverse transcriptase (BD-Biosciences) in the presence of amino allyl dNTP (Sigma), oligo (dT)_18_, and 0.5 μl spiking RNA mix (0.25 ng of each 10-Alien mRNA; Stratagene). The resulting cDNA was cleaned-up using the Qiagen PCR purification kit (Qiagen) and coupled with the corresponding fluorescent dye Cy3 or Cy5 (Amersham). The fluorescent labeled cDNA was purified using the Qiagen PCR purification kit. Microarray slides were processed and prehybridized as described (Hu et al., 2003). The fluorescent labeled cDNA was then resuspended in 15 μl hybridization buffer plus 1 μl of oligo poly(dA)_50_. The probe was then denatured, pre incubated at 42°C for 20 min and applied to the microarray placed in a waterproof hybridization chamber (AHCXD. 2.5 mm deep: Telechem) and covered with a lifter slip (1 mm, 22LX25; Erie Scientific Co, Portsmouth, NH). Hybridization was carried out in a 42°C water bath for 18 h. After hybridization slides were washed followed by 10 s dip in DyeSaver (Genisphere Inc., Hatfield, PA). Microarray hybridizations for each treatment or tissue were performed as a set of at least two independent biologically replicate experiments with corresponding untreated controls for each treatment. Assuming that data analysis using two biological replicate experiments would reduce false differential gene expression and experimental variations to <0.05% as suggested (Schenk et al., 2000).

### Microarray data analyses

Slides were scanned by GenePix 4100A (Axon™ Instruments, Union City, CA). The data was extracted with Axon GenePix Pro 5 image analysis software. The spot sizes and intensities quantified by the software and automatically flagged spot qualities were followed manual examination. Abnormal shape spots or spots with high local background or spots that were quantified due to false intensity caused by dust were flagged bad and discarded. Various methods for the normalization of the intensity values from the two channels were performed, but global normalization fit well to all experimental treatments and was used for data analysis. Data points with background subtracted median intensity signals <60% +2 SD above the overall background intensity in both channels were discarded. Ratios were calculated using Cy3 (treated)/Cy5 (mock or untreated) and converted to log_2_ ratios by the software. Further data analyses such as average of two experimental replicates, significance of induction and suppression, sorting and counting, graphical representation and cluster analysis, all were performed using data analysis software Acuity 4 (Axon) and Microsoft Excel.

### Statistical analysis of microarray data

The microarray data from 60 microarrays was presented in a single Excel Dataset (Supplemental Table 1). Each microarray was generated by one of 2 experimental runs by 2 treatments by 3 time points by 5 biological repetitions by treatment-control pairs. Treatment RNA was stained with Cy3(532) dye and its corresponding control RNA was stained with Cy5(635) dye on the same microarray. Each microarray was printed with 1287 experimental probes of which 26 probes were duplicated. There were 1261 unique two color probes per microarray.

The Cy3 and Cy5 optical intensity measures per probe and their ratio (Cy3/Cy5) were tested for statistical symmetry using the Box-Cox procedure. The results indicated that a log transformation of the intensities or their ratio was indicated. Since all experimental treatments used Cy3 and all control treatments Cy5, the log_2_ (Cy3/Cy5) was chosen as the dependent variable for statistical modeling. Side by side Box-plots of the array/dye combinations was run to verify the both the completeness and plausibility of the input microarray data. A parallel modeling system was developed with Cy3 and Cy5 modeled separately. A comparison of the modeling results indicated that ratio model was better than the separate dye model at fitting the observed data. The data was then modeled using a two stage mixed linear model. This is an adaptation and extension of an analysis system proposed by Kerr et al. (2000), Wolfinger et al. (2001), and Efron et al. (2001).

In the first stage the variability of across array hybridization and dye binding was removed by the model.

$${\text{log}}_{2}(\text{Cy3}/\text{Cy5})=\beta_{0}+\beta_{1}\text{M}\_\text{Array}+b_{0}+\varepsilon$$

M_Array is the unique microarray identifier.

In the second stage the variability of residual log-ratio values by treatment and time were modeled by unique probe ID.

$$e=\beta_{\iota 0}+\beta_{\iota 1}\text{Trt}+\beta_{\iota 2}\text{Time}+\beta_{\iota 3}{\text{Trt}}^{*}\text{Time}+b_{i0}+\varepsilon^{*}$$

Trt is the treatment (Psyr or Sp)
Time is the time post-treatment (1, 6, 24 h)

Testing for statistically significant differences was done with an F-test and expression levels significantly different from zero were tested with a *t*-tests for each unique probe. Differential expression was tested for between Trt, Time, and Trt^*^Time. These *p*-values were adjusted to correct for multiple hypothesis testing using the Tukey–Kramer procedure. These data were then written to an output Excel file for review by the biologists. The residuals from the second model were also tested for patterns in the residuals. The model fit the data well, and there were no patterns that indicate a problem with the model.

The *p*-values from the *F*-test of Trt^*^Time were then used to determine the number of probes that meet the FDR criteria with α = 0.05. There are 771 probes that meet the FDR criteria. These are the probes that show a statistically significantly difference between treatment and control by time from among the 1261 unique probes on the microarray. Let Y_ID,Trt^*^_Time be one of the 6 values for differential expression for each of these probe IDs across treatment by time. These are the values that will be analyzed further with principal components and clustering.

The clustering analysis was performed using the covariance matrix and the 6 expected treatment values per probe. Standardized canonical covariance coefficients were computed from these data. The 769 observations without missing values were then clustered using an agglomerative hierarchical algorithm and using Wards method to minimize the variance within each cluster. There were potentially stable clustering levels at 3, 6, 9, and 12 clusters. Given the structure of the study with 3 time courses, two treatments, and treatment and control, 6 clusters were chosen for closer examination of differential expression between treatment and control.

### Metabolite extraction

All plant samples were freeze-dried and ground in 1 mL centrifuge tubes. Samples masses are approximately 15 mg—exact masses are recorded in data spreadsheet. To each centrifuge tube was added 1.00 mL 50/50 methanol/water containing 13.3 μg/mL of each of the internal standards (a series of alkyl 4-hydroxybenzoates). The tray of sample tubes was wrapped in foil and stored at 9°C for 24 h, at which point the supernatant was transferred to autosampler vials for LC-MS analysis.

### Metabolite chromatographic separation

All samples were analyzed on a Shimadzu (Kyoto, Japan) SCL-10ADvp HPLC system with a Thermo (Bellefonte, PA) Betabasic C18 (150 × 1 mm; 5 μm particle size) reverse phase column connected directly to the mass spectrometer ion source. Chromatographic separation was achieved using elution solvents A = 0.15% v/v aqueous formic acid, B = methanol. Initial conditions were 1% B and the solvent gradient began at 0 min and ramped to 100% B over 37 min. It was then held at 100% B for an additional 6 min, after which the composition returned to the initial condition. The flow rate was approximately 100 μL/min and each injection consisted of 10 μL plant extract.

### Metabolite mass spectrometric analysis

The HPLC was coupled to a Micromass (Manchester, UK) Quattro II mass spectrometer. The instrument was equipped with an electrospray ionization source, and plant extracts were analyzed separately in both the negative and positive modes. The mass spectrometer was operated with alternating cone voltage in parallel data acquisition channels (Bateman et al., 2007) such that two spectra were acquired at every time point—one at low cone voltage (20 V) and one at high cone voltage (75 V). In this way, in-source collision induced dissociation (CID) was achieved resulting in both molecular ions and the corresponding fragment ions in separate spectra. For negative mode experiments, the scan range was *m/z* 100–1000, whereas for positive mode experiments, the scan range was *m/z* 100–1500. The source block temperature was 100°C, and the source capillary voltage was −2.5 kV (negative-ion mode) or +3.0 kV (positive-ion mode).

### Metabolite data processing

All chromatograms were processed using Waters MassLynx software, v.4.0 using the QuanLynx routine. Using the MassLynx method editor, selected extracted ion chromatograms (XICs) were integrated and that peak area information is tabulated in the Excel spreadsheet. Particular attention was paid to the glucosinolates and polyphenolics. Each raw peak area was then adjusted for sample size and to the nearest-eluting internal standard. The adjusted data were then normalized to 100. Both low and high cone voltage spectra were examined for each metabolite peak integrated to provide confirmatory evidence for metabolite identity. When helpful, the low cone voltage spectra with the background subtracted are also included as well as expanded spectra showing the pseudomolecular ions for the glucosinolates.

### Statistical analysis of mass spectrometry metabolite data

The mass spectrometry metabolite data were generated by one of 2 experimental runs by 2 treatments by 2 control by 1 time point by 8 biological repetitions for a total of 64 analyses. Each run collected quantification data for 33 metabolite fractions (Supplemental Table 2). The quantification data was tested for statistical symmetry using the Box-Cox procedure. The results indicated that a log transformation of the integrated area was indicated. The distribution of the integrated areas was examined. Metabolites that were only present under some experimental conditions were different from metabolites that were in all samples but in different relative amounts. Metabolites that were not always present need to be analyzed as present/absent in a dichotomous fashion. Metabolites that were in nearly all samples have a continuous distribution and, once transformed, can be analyzed using a mixed linear model. There were not enough observations to model the dichotomous metabolites.

The following linear mixed model was fit to the continuous data.

$$\begin{array}{l} \log_{2}(\text{Fraction})=\beta_{0}+\beta_{1}\text{Exp}+\beta_{2}\text{Trt}+\beta_{3}\text{TC}+\beta_{4}Trt^{*}TC\\ \;+b_{0}+\varepsilon \end{array}$$

Exp. is experiment (1 or 2).
Trt is treatment (Psyr or Sp).
TC is treatment/control (T or C).

Testing for statistically significant differences was done with an *F*-test and expression levels significantly different from zero were tested with a *t*-tests for each unique metabolite. Differential expression was tested for between Trt, TC, and Trt^*^TC. These *p*-values were adjusted to correct for multiple hypothesis testing using the Tukey–Kramer procedure. These data were then written to an output Excel file for review by the biologists. The residuals from the model were also tested for patterns in the residuals. The model fit the data well.

### Gene expression profiling in response to separate caterpillar and pathogen treatments

This experiment compared the effects of insect and pathogen attack on the expression of “stress” genes in *Arabidopsis thaliana* (ecotype Columbia), and statistically related changes in gene expression to changes in metabolites and insect performance. There were four plant treatments: (1) infection with *Pseudomonas syringae* pv. *tomato* [avirulent strain DC300 (avrRpm1)], (2) infiltration control, (3) feeding by *S. exigua* caterpillars, and (4) caterpillar cage control. This kind of experiment has two significant design constraints. First, we wanted to collect only damaged leaves to examine local (and not systemic) responses, so we had to “wrangle” caterpillars—i.e., move them around from leaf to leaf to prevent them from eating entire leaves and to distribute their feeding among several leaves. Second, we wanted to take samples at specific, known time intervals after damage so we could identify genes expressed early, middle, and late in the response. This required keeping feeding to a short, specific time interval (~1 h). All stages of the experiment were conducted at 25°C.

Leaves damaged (10–30% damage) by caterpillars or infiltrated with pathogens and their respective controls, as described above, were sampled at 1, 6, and 24 h after treatment for gene expression, and 48 h for metabolite analysis because glucosinolates (GS) take 24–48 h to accumulate. Since infiltration of all replicates in a treatment/time group took up to 20 min and caterpillar feeding up to 1 h, we took the midpoint as our starting treatment time. We treated and harvested 6 leaves per plant. To produce 5 biological replicates with sufficient RNA for microarrays (+100 μg RNA per sample), we treated 20 plants and pooled leaves from 4 plants for each biological replicate. To produce 8 biological replicates with sufficient GS for chromatographic analyses, we treated 32 plants and pooled leaves from 4 plants for each biological replicate. Details of the RNA extraction and microarray analysis, and metabolite extraction and analysis, are provided in more detail below.

To visualize relationships among the treatments based on their similarity in stress gene expression, we clustered treatments using expression data from 1236 unique genes. Our goal was to explore the potential relationships between the transcriptional responses we found in our insect and bacterial treatments with the responses found in response to other stressors (e.g., drought, temperature, oxidative stress, etc.). We followed procedures developed by Eisen et al. (1998) using the TIGR Multiexperiment Viewer v4.8.1 (http://www.tm4.org/mev.html) to explore 8 different approaches to create hierarchical clusters of gene expression patterns, using correlation and covariance data and alternative linkage procedures. We also calculated the covariance matrix for the expression data and viewed the resulting Eigen values to estimate the number of clusters to consider. The analysis indicated that 10 or 11 clusters would explain about 85% of the variance in the matrix with minor gains from including additional clusters.

### Pathogen bioassay with caterpillar pretreatment

This experiment evaluated the impact of caterpillar feeding on subsequent susceptibility of Arabidopsis to attack by a virulent (*Pst* DC3000) and avirulent Pst DC3000 (*avrRpm1*) *P. syringae* pv. *tomato* strain. The virulent strain lacks the AvrRpm1 effector that cleaves RIN4 from Arabidopsis membranes to block RPM1 activation (Kim et al., 2005). All stages of the experiment were conducted at 25°C. Pre-treatment consisted of feeding by second-instar *S. exigua* caterpillars for 24 h and a cage control (no-insect in cage). Plants were then infiltrated by syringe at a titer of 5 × 10^7^ CFU/ml in 10 mM MgSO_4_of the virulent strain, the avirulent strain, or an inoculation control of 10 mM MgSO_4_. In total, 36 plants were treated, with 6 plants per treatment with leaves were sampled daily for 3 days after inoculation for use in monitoring pathogen growth and expression of *PR1*.

### Pathogen growth assay

Colony growth was measured in leaf disks removed from infiltrated leaves with a #2 cork borer (6.25 mm). Disks were ground in 10 mM MgSO_4_, and 100 μl of the appropriate dilutions was spread on petri plates containing KB growth media. After 3 days, the number of colonies on the plates was counted and used to calculate the number of CFU/cm^2^ of leaf area.

### RNA extraction and quantification of *PR1*

Expression of *PR1* was determined in total RNA extracted using a modified Trizol method, treated with DNase, and reverse transcribed. Expression of *PR1* and *18S* in the resulting cDNA was examined by Real-Time PCR using the following primers for PR1 and 18S, respectively; 5′-GTGGGTTAGCGAGAAGGCTA-3′ and 5′-CATCCTGCATATGATGCTCCT-3′, 5′-CGGCTACCACATCCAAGGAA-3′ and 5′-TGCTGGCACCAGACTTGCCCTC-3′. ΔC_T_ was calculated by subtracting the C_T_ of the reference gene (*18S*) from the C_T_ of *PR1*.

## Results

### Effect of caterpillar or pathogen attack on gene expression and metabolites

We compared the response of Arabidopsis to either a caterpillar or an avirulent pathogen by profiling changes in gene expression and metabolites. We used a custom microarray of genes differentially expressed in response to a wide range of biotic and abiotic stressors (Mahalingam et al., 2003) and sampled at 1, 6, and 24 h after attack.

**Figure 1:**
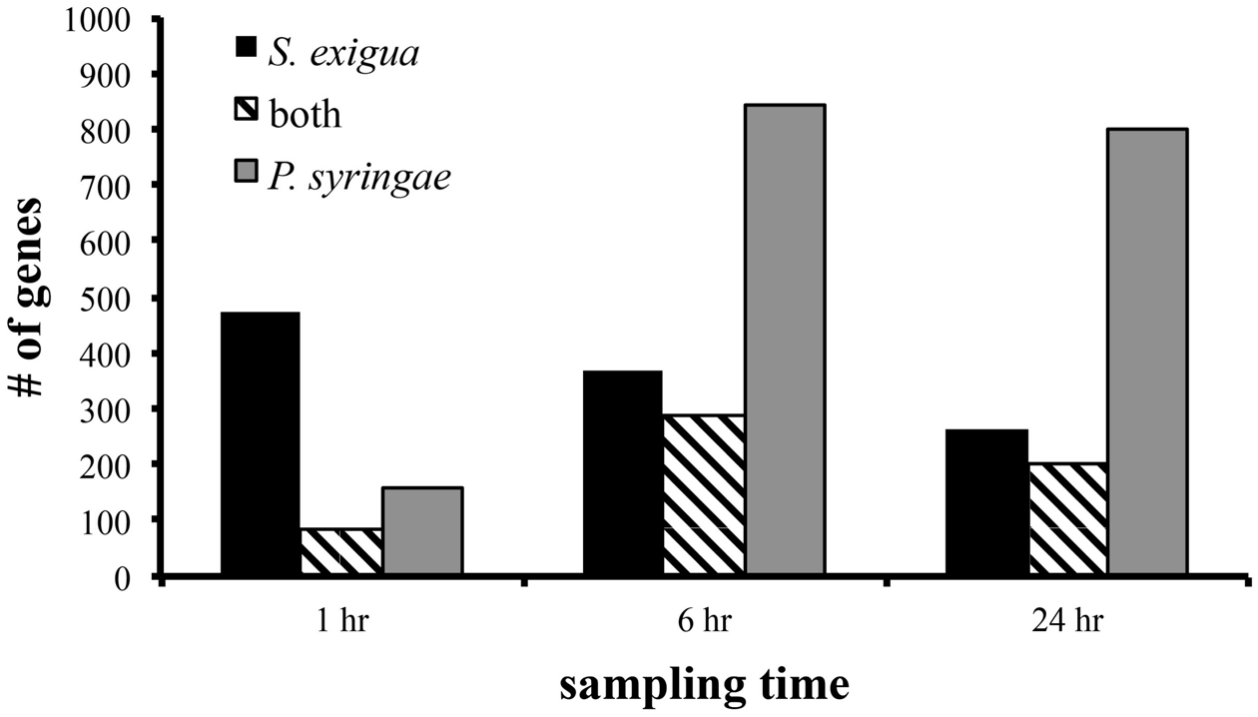
**Number of genes differentially expressed by Arabidopsis in response to attack by *S. exigua* and avirulent *P. syringae* pv. tomato.** Only those genes whose expression was statistically significantly altered at *p* < 0.001 level were included in this summary.

The number of statistically significant changes in the expression of genes in Arabidopsis leaves differed with the treatments and sampling time (Figure 1). Response of genes differentially expressed in response to caterpillar feeding was rapid, occurring within 1 h of feeding, and was lower at 6 h and declined further at 24 h. In contrast, response of genes differentially expressed in response to pathogen infection was low at 1 h but was higher at 6 and 24 h (Figure 1). Pathogen treatment caused almost twice as many genes to change in expression than caterpillar treatment overall. There was substantial overlap in the differentially expressed genes for each treatment, ranging from a low of 84 genes at 1 h to 287 and 203 genes at 6 and 24 h, respectively (Figure 1). A GO analysis of those genes did not reveal statistically significant enrichment of any functional categories.

**Figure 2:**
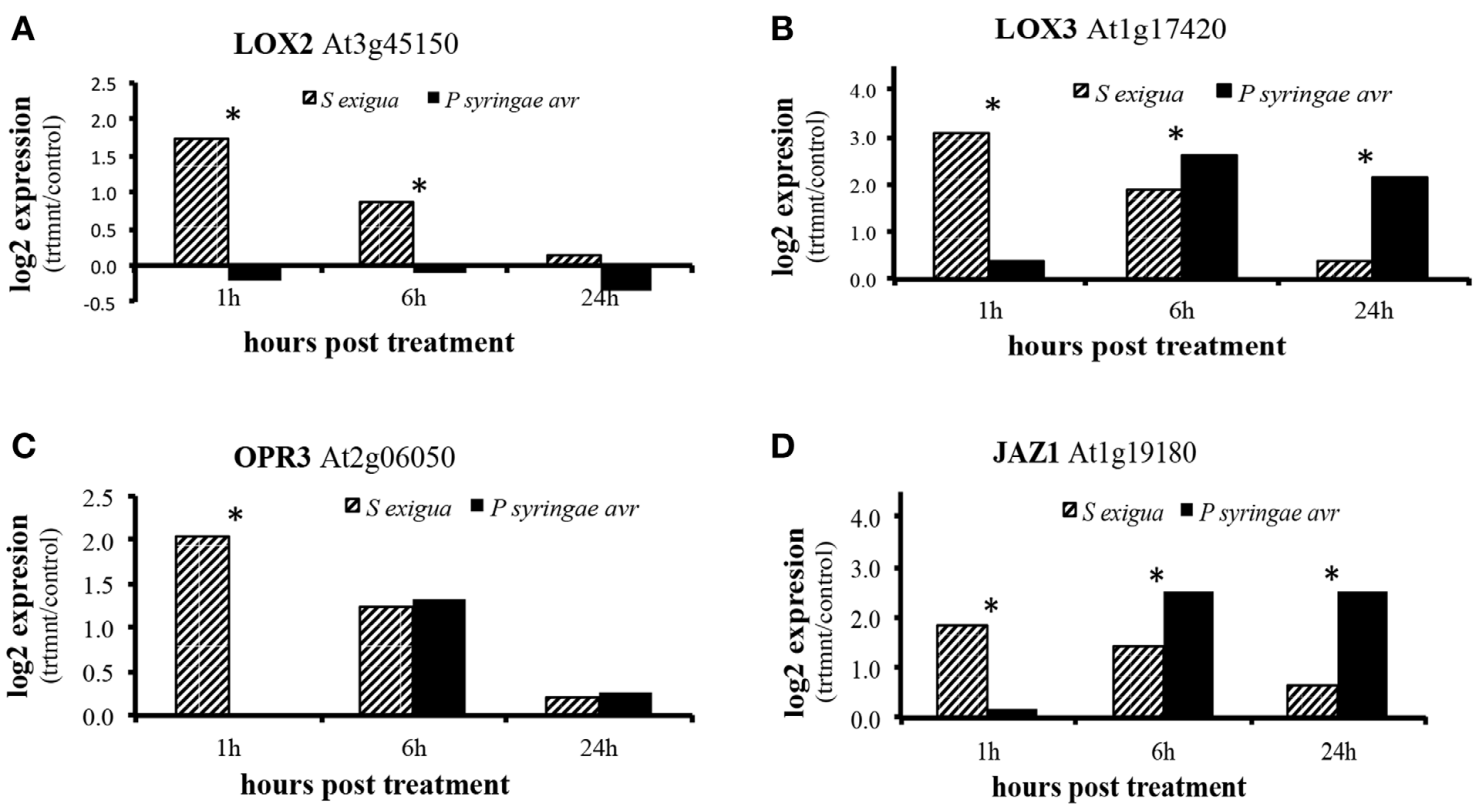
**Differential expression of genes associated with jasmonic acid signaling in Arabidopsis response to attack by *S. exigua* and avirulent *P. syringe* pv. *tomato*. (A)** *LOX2* (lipoxygenase 2; At3g45140) **(B)** *LOX3* (lipoxygenase 3; At1g17420) **(C)** *JAZ1* (jasmonate-ZIM domain; At1g19180) **(D)** *OPR3* (12-oxo-phytodienoic acid reductase 3; At2g06050). *N* = 5 and asterisks indicate statistically significant increase in log_2_ expression above control values at *p* < 0.05.

Expression of several genes whose products are involved in JA biosynthesis and signaling were up-regulated by caterpillar feeding (Figure 2). These include lipoxygenase 2 (*LOX2*), lipoxygenase 3 (*LO*X3), 12-oxo-phytodienoic acid reductase (*OPR3*), and jasmonate–ZIM-domain protein 1 (*JAZ1*), all of whose transcripts were upregulated at 1 and 6 h and returned to near control levels by 24 h. Surprisingly, transcripts for LOX3, OPR3, and JAZ1 were also up-regulated by the pathogen but at later time points than by the caterpillars. None were differentially expressed in response to the pathogen at 1 h, but OPR3 transcripts were induced only at 6 h while transcripts for LOX3 and JAZ1 transcripts were induced at 6 and 24 h. There was no differential expression of LOX2 in response to pathogen attack.

**Figure 3:**
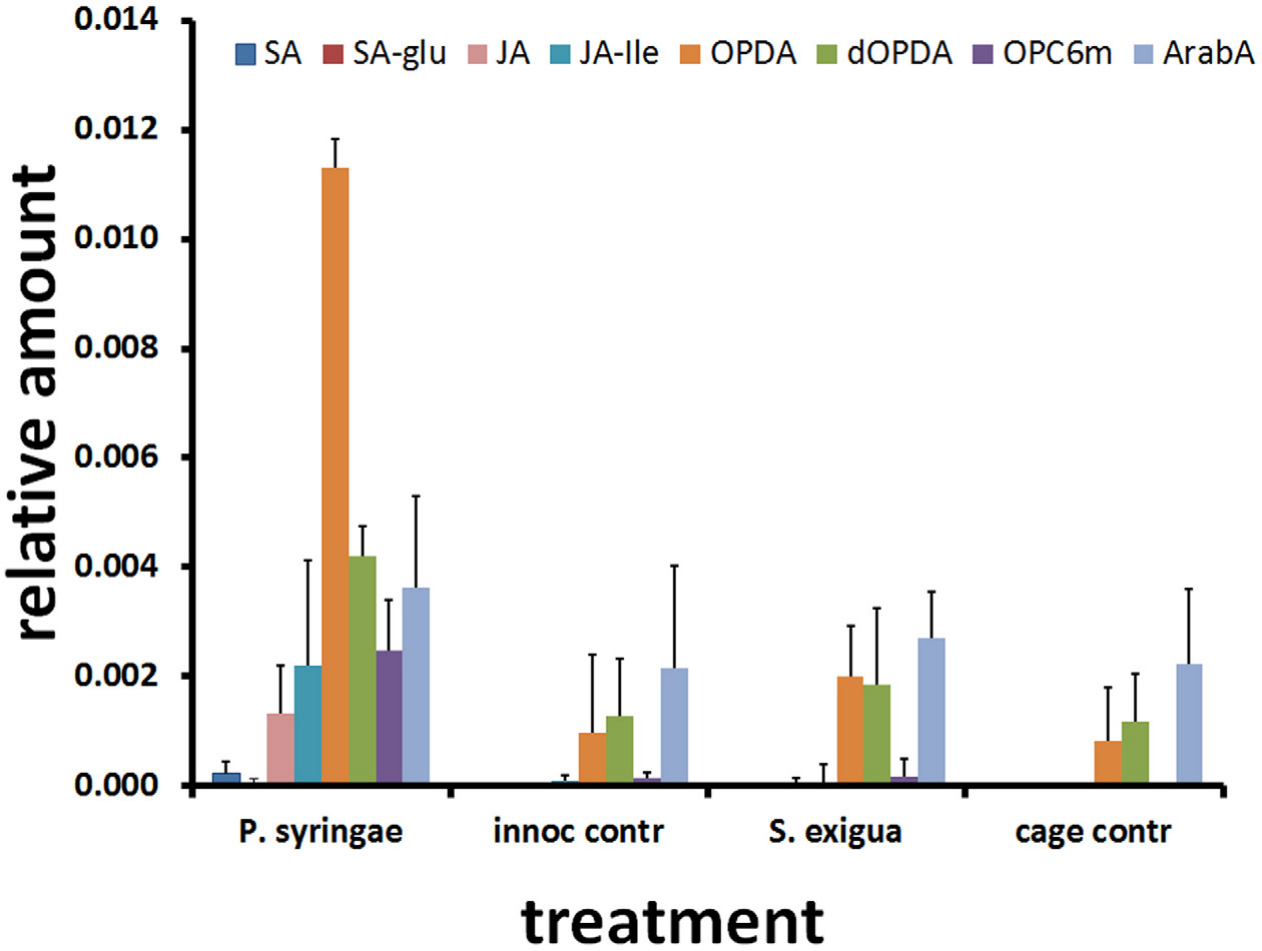
**Arabidopsis metabolite responses to attack by *S. exigua* and *P. syringae* and their respective controls.** Average and SD values of SA and JA-related hormones (*n* = 8) 48 h after treatment. SA, salicylic acid; SA-glu, salicylic acid glucoside; JA, jasmonic acid; JA-ile, jasmonoyl isoleucine; OPDA, oxophytodienoic acid; dOPDA, dinor oxophytodienoic acid; OPC6m, 3-oxo-2-(2#-[Z]-pentenyl)-cyclopentane-1-hexanoic acid malate; ArabA, Arabidopside A.

**Figure 4:**
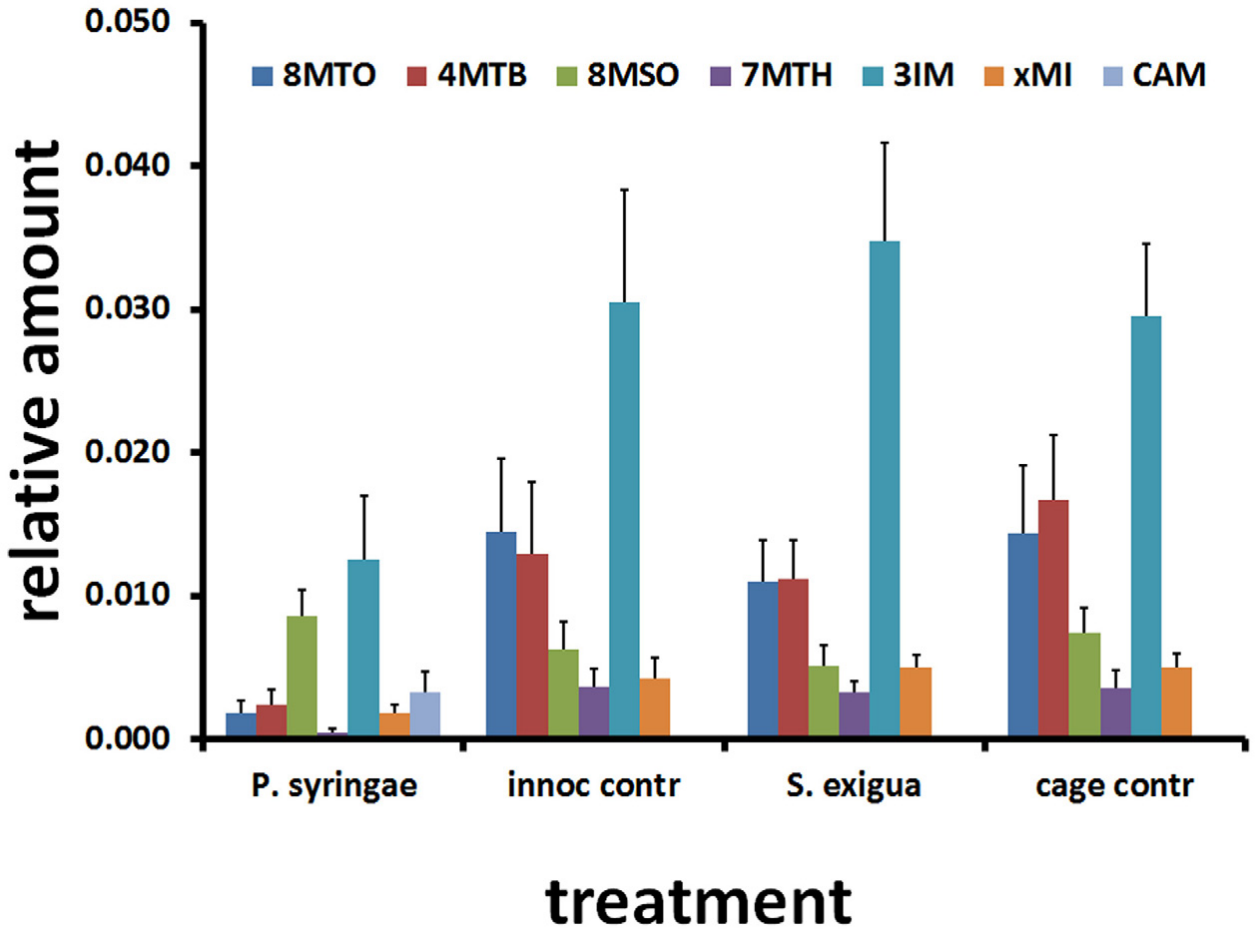
**Arabidopsis metabolite responses to attack by *S. exigua* and *P. syringae* and their respective controls.** Average and SD (*N* = 8) of relative amount of glucosinolates and camalexin 48 h after treatment. 8MTO, 8-methylthiooctyl glucosinolate; 4MTB, 4-methylthiobutyl glucosinolate (glucoerucin); 8MSOO, 8-methylsulfinyloctyl glucosinolate (glucohirsutin); 7MTH, 7-methylthiohexyl glucosinolate; 3IM, 3-indolmethyl glucosinolate (glucobrassicin); xMI, methoxyindolyl glucosinolate; CAM, camalexin, 3-thiazol-2′yl-indole.

We then examined whether differences between treatments in gene expression were reflected in differences in hormones and glucosinolates in leaves sampled 2 days after attack (Figures 3, 4). This later time point was chosen because it provides enough time for the glucosinolate (GS) response to develop fully (data not shown). There was no induction of JA metabolites by caterpillars still detectable at 48 h. Predictably, SA and SA glucoside (SAG) were absent in the caterpillar treatment, cage control, and inoculation control but were present in the pathogen treatment at low levels. However, levels of JA metabolites were as high—or higher—in the pathogen treatment than the caterpillar treatment, including JA, JA-Ile, and, especially, 12-oxo-phytodienoic acid (OPDA).

The caterpillar and pathogen treatments induced contrasting patterns in the expression of genes involved in glucosinolate biosynthesis and activity (Table 1). Caterpillars upregulated many genes involved in glucosinolate biosynthesis, whereas the avirulent pathogen downregulated them. Compared to the pathogen treatment, caterpillars elicited greater expression of genes involved in production of aliphatic glucosinolates, including methylthioalkylmalate synthase 1 (MAM1), methylthioalkylmalate synthase-like (MAML), two thiohydroximate *S*-glucosyltransferases (UGT74B1, UGT74C1), two cytochrome P450s (CYP79F1, CYP83A1), and a 2-oxoglutarate-dependent dioxygenase (AOP2). Caterpillars also elicited greater expression of genes involved in indolyl glucosinolate production, including three cytochrome P450s (CYP79B1, CYP79B2, CYP79B3). The only glucosinolate-associated genes whose expression was downregulated by the caterpillar were two myrosinase genes, thioglucoside glucohydrolase 1 and 2 (TGG1, TGG2).

**Table 1:**
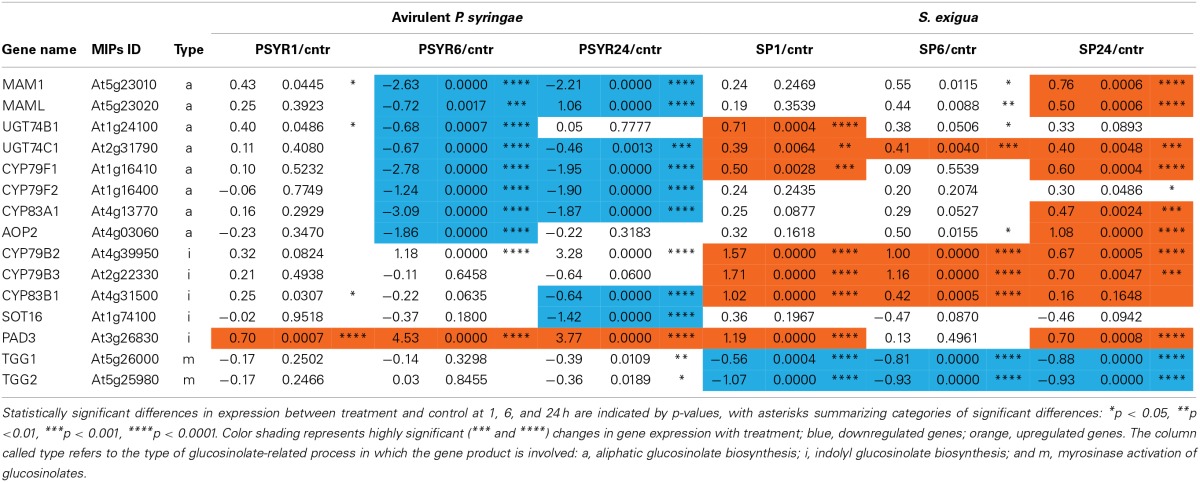
**Expression (log_2_ of treatment/control) of Arabidopsis genes involved in glucosinolate biosynthesis and activity that are differentially expressed in response to attack by *S. exigua* and avirulent *P. syringe* pv. *tomato***.

Statistically significant differences in expression between treatment and control at 1, 6, and 24 h are indicated by p-values, with asterisks summarizing categories of significant differences: ^*^p < 0.05, ^**^p <0.01, ^***^p < 0.001, ^****^p < 0.0001. Color shading represents highly significant (^***^ and ^****^) changes in gene expression with treatment; blue, downregulated genes; orange, upregulated genes. The column called type refers to the type of glucosinolate-related process in which the gene product is involved: a, aliphatic glucosinolate biosynthesis; i, indolyl glucosinolate biosynthesis; and m, myrosinase activation of glucosinolates.

In contrast, the avirulent pathogen downregulated many genes involved in production of aliphatic glucosinolates, including MAM1, MAMl, UGT74B1, UGT74C1, CYP79F1, CYP79F2, CYP83A1, and AOP2. Pathogen treatment also downregulated genes involved in indolyl glucosinolate production, including CYP83B1 and a desulfoglucosinolate sulfotransferase (SOT16). The only gene whose expression was altered by both caterpillar and pathogen in the same direction was phytoalexin-deficient 3 (*PAD3*), which encodes a cytochrome P450 that catalyzes the last step in the biosynthesis of the indole-derived phytoalexin camalexin.

The caterpillar and pathogen treatments induced different patterns of glucosinolate metabolites (Figure 4). Consistent with its down regulation of glucosinolate biosynthesis genes, the pathogen treatment had lower levels of most aliphatic and indolyl glucosinolates at 48 h compared to the caterpillar treatment. Camalexin was the only exception to this pattern and was only in the pathogen treatment, consistent with the higher expression levels of the *PAD3* gene whose product comprises the final step in camalexin biosynthesis.

**Figure 5:**
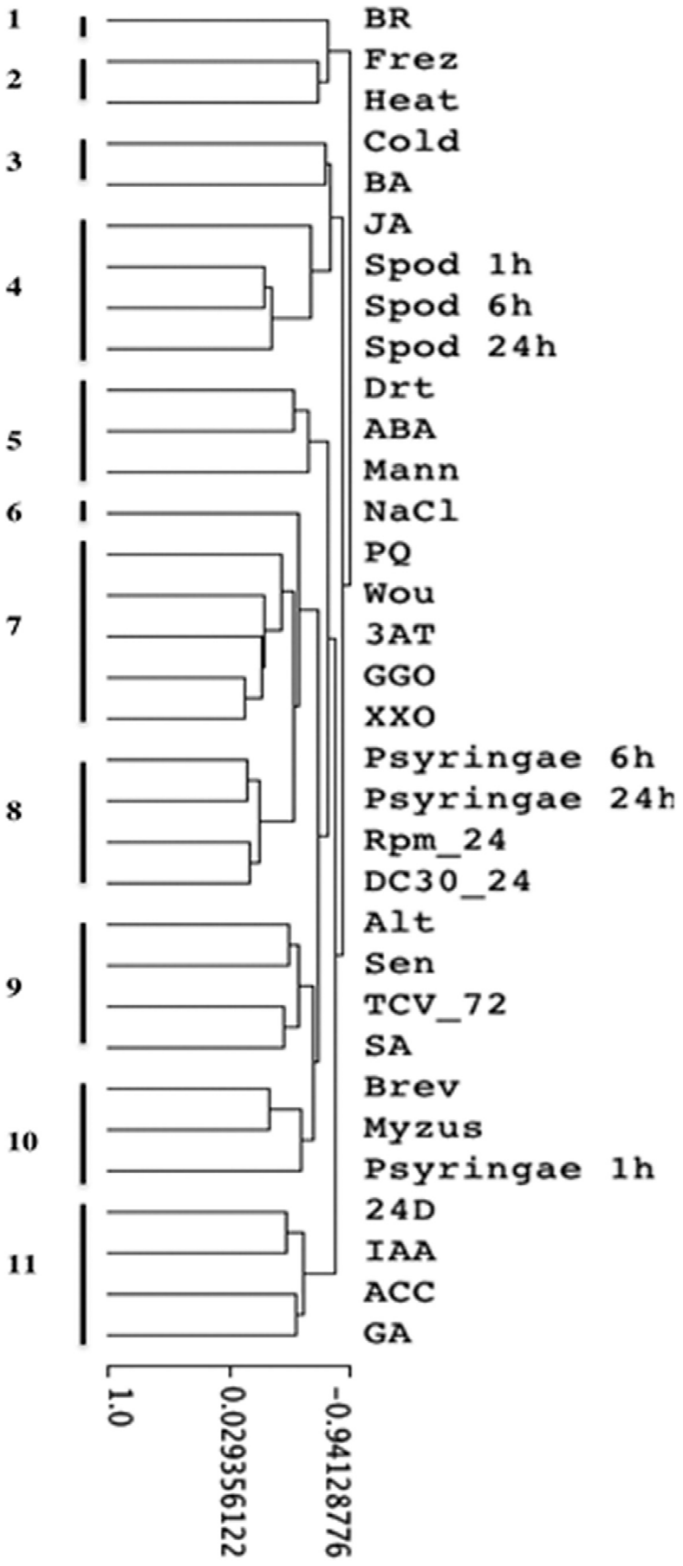
**Hierarchical clustering of stress array expression data by treatment, including results of insect feeding and pathogen infection experiments.** Eleven clusters were evident: 1, response to brassinosteroid; 2, Freeze/heat stress responses; 3, Cold/Cytokinin stress responses; 4, responses to three insect treatments/jasmonate signaling; 5, Drought stress responses; 6, NaCl stress response; 7, responses to oxidative stresses/wounding; 8, late responses to *P. syringae* and responses to Rpm24, DC30 bacteria; 9, salicylate-mediated responses to microbes; 10, responses to aphids and early response to *P. syringae*; 11, suite of responses to 4 hormones. Key to treatments: BR, brassinosteroid; Frez, freezing; heat, heat; cold, cold; BA, 50 μM 6-benzyladenine (synthetic cytokinin); JA, 50 μM jasmonic acid; spod, *Spodoptera exigua*; drt, drought; ABA, 50 μM abscisic acid; Mann, 300 mM mannitol; NaCl, 300 mM NaCl; PQ, paraquat; Wou, wound; 3AT, 4 mM 3-amino-1,2,4-triazole; GGO, glucose-glucose oxidase; XXO, 2 mMxanthine-xanthine oxidase; Psyringae, *Pseudomonas syringae* pv. tomato; RPM, *Pseudomonas syringae* pv. tomato DC3000 (*avrRpm1*); DC30, *P. syringae* pv. tomato DC3000; Alt, *Alternaria brassicola*; Sen, senescence; TCV_72, turnip crinkle virus; SA, 2 mM salicylic acid; Brev, *Brevicoryne brassicae*; Myzus, *Myzus persicae*; 24D, 50 μM 2,4-dichlorophenoxyacetic acid (synthetic auxin); IAA, 50 μM indoleacetic acid; ACC, 50 μM 1-aminosyclopropane-1-carboxylic acid (ethylene precursor); GA, 50 μM gibberellic acid. Details of the experimental treatments are found in Mahalingam et al. (2003).

Clustering approaches produced 11 obvious clusters, most of which were found in all approaches explored. The best-defined clusters emerged using Pearson correlation coefficients and average linkage values. Eleven clusters were evident (Figure 5): 1 = response to brassinosteroid treatment, 2 = freeze/heat stress responses, 3 = cold/cytokinin stress responses, 4 = responses to three insect treatments/jasmonate signaling, 5 = drought stress responses, 6 = sodium chloride stress response, 7 = responses to oxidative stresses/wounding, 8 = late responses to *P. syringae* and to virulent Rpm24, DC3000 bacteria, 9 = salicylate-mediated responses to microbes, 10 = responses to aphids and early response to *P. syringae*, 11 = suite of responses to 4 hormones.

Most of the clusters could be interpreted biologically. As one would expect, oxidative stress treatments clustered together, although response to wounding was included in that cluster (cluster #7). Salicylate-mediated responses to microbes and SA treatments formed another cluster (#9). Two singleton clusters were seen: responses to brassinosteroids (#1) and responses to NaCl (#6).

The focus of this clustering was on the similarity of transcriptional responses in our experiments with *P. syringae* and *S. exigua* to the other stress responses. Not surprisingly, response profiles in experiments with *P. syringae* were most like those seen to other *P. syringae* genotypes in developing the stress array (Ps DC3000, Ps RPM24). This result confirms the utility of using the array to describe and understand new transcriptional results. Interestingly, the earliest response to our *P. syringae* inoculations (at 1 h post-inoculation) clustered with responses to two aphid species, rather than with other bacteria (#10).

Responses to the caterpillar at 3 time points grouped together in a cluster that also included responses to JA (cluster #4). Interestingly, response to wounding was not found in that cluster (but clustered instead with oxidative stress responses, #7). The cluster most closely joined to the caterpillar group was characterized by responses to cold and cytokinins (#3).

### Effect of pre-treatment with caterpillars on susceptibility to avirulent and virulent *p. syringae*

**Figure 6:**
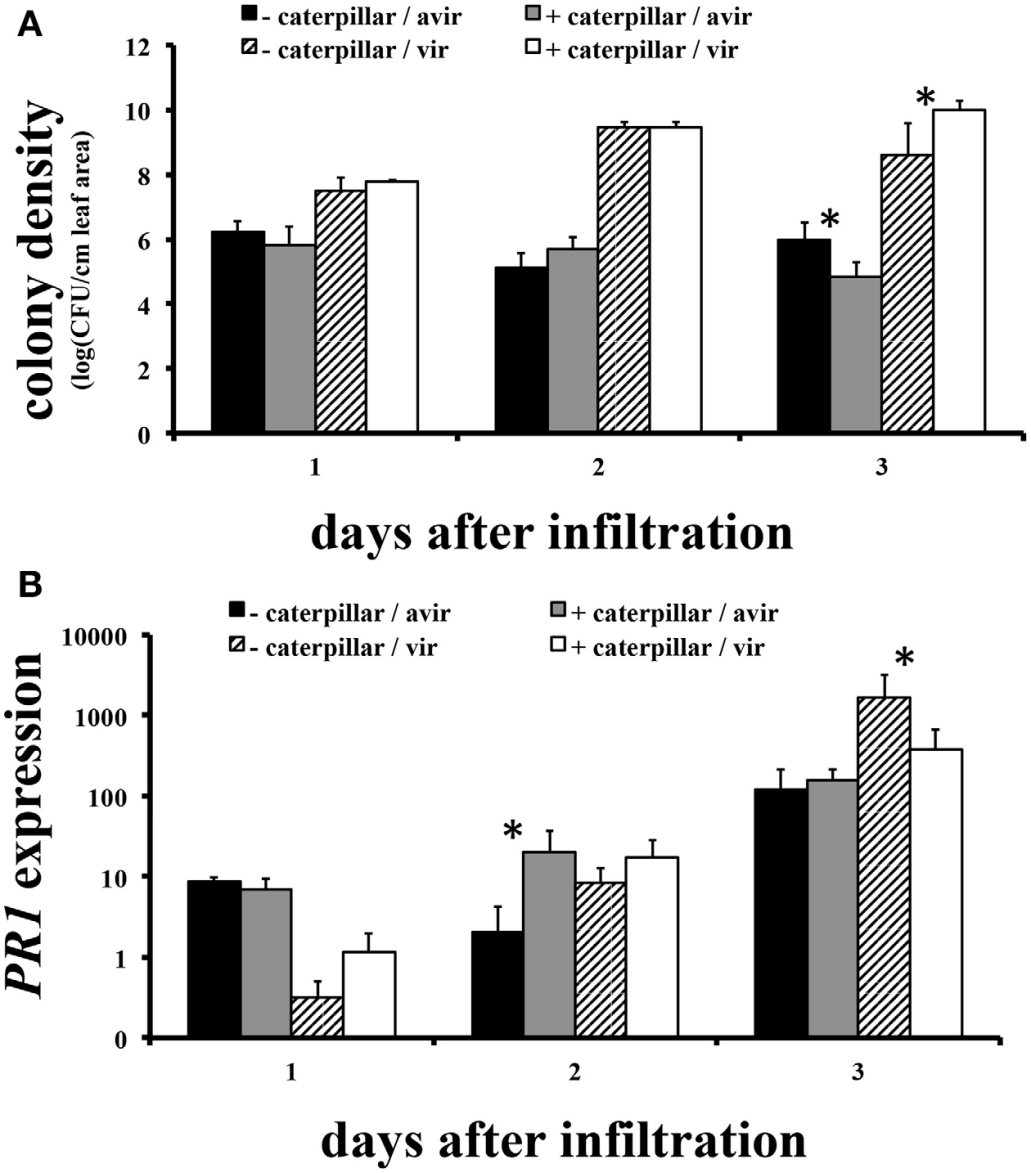
**Effect of pre-treatment with *S. exigua* feeding on susceptibility of Arabidopsis to *P. syringe* pv. *tomato* virulent and avirulent strains and the expression of Arabidopsis *PR1*. (A)** Bacterial population growth. *N* = 4–6. **(B)** Relative *PR1* gene expression (treatment/control) *N* = 3. ^*^Indicates a statistically significant effect of caterpillar pretreatment at *p* < 0.05.

Transcriptional data suggested that attack by the herbivore might affect subsequent pathogen performance. To examine this possibility, we exposed Arabidopsis plants to 24 h of *S. exigua* feeding and then measured the growth of avirulent and virulent *P. syringae* colonies on insect-treated and control plants (Figure 6). Colonies of avirulent *P. syringae* failed to increase over 2 days post-inoculation (pi) in both insect treatments, while colony numbers were significantly depressed only on caterpillar-treated plants by day 3pi. At the same time, colonies of virulent *P. syringae* grew over the first 2 days as expected, but continued to increase on day 3pi only on caterpillar-treated plants (Figure 6A).

We examined the expression in insect-treated and control plants of PR1, a commonly-used indicator of transcriptional responses to infection. The main impact of insect attack on PR1 levels was to alter the timing of response to avirulent and virulent bacteria. Insect-treated plants responded to avirulent bacteria with significantly elevated PR1 expression only on day 2pi, a day before avirulent colony growth on those plants exceeded controls. Insect-treated plants responded to virulent *P. syringae* with PR1 expression elevated above controls as well, but not until day 3pi, the day on which bacterial colony growth was suppressed on insect-treated plants (Figure 6B).

## Discussion

### Effect of caterpillar or pathogen attack on gene expression and metabolites

Feeding by *S. exigua* induced a rapid response in gene expression that waned by 24 h, whereas response to avirulent *P. syringae* was weak at 1 h but increased substantially at 6 h and remained strong at 24 h. These differences in the timing of the maximum gene expression responses by Arabidopsis to the herbivore and pathogen may have resulted from the duration and maxima of membrane depolarization. Bricchi et al. (2012) found that the maximum amount of gene expression changes in Arabidopsis occurred much later in response to avirulent *P. syringae* than in response to the caterpillar *S. littoralis*, coincident with the timing of maximum membrane depolarization by each treatment.

Temporal changes in gene expression observed in response to *P. syringae* were evident in the hierarchical clustering analysis. Whereas later responses to *P. syringae* clustered together and with those reported for other *P. syringae* genotypes, the earliest response to *P. syringae* clustered with aphids. There are other examples of similarities in plant responses to aphids and bacteria (Goggin, 2007), but it is not clear why only the earliest responses to *P. syringae* clustered with responses to aphids. That result suggests some interesting questions about similarity between elicitors in those insects and *P. syringae*.

There was substantial overlap in the differentially expressed genes for each treatment, ranging from a low of 84 genes at 1 h to 287 and 203 genes at 6 and 24 h, respectively. Others have reported overlap in differential gene expression by Arabidopsis in response to microbes and herbivores, but the degree of similarity varies widely among studies. De Vos et al. (2005) found approximately half of the genes whose expression was induced by avirulent *P. syringae* were also induced by the caterpillar *P. rapae* 12 and 24 h after treatment, whereas Bricchi et al. (2012) found less than a quarter of the genes whose expression was induced by avirulent *P. syringae* were also induced by *S. littoralis*. Given the variation in overlap of differentially expressed genes in our study, which ranged from 18–77% depending on the sampling time, it should not be surprising to find substantial variation within and between studies. This observation should encourage caution in interpretation of datasets lacking a time course of sampling and those derived using different species with possibly different elicitor levels, or both.

Not surprisingly, transcripts for several genes whose products are involved in JA biosynthesis were upregulated by caterpillar feeding at 1 and 6 h, consistent with other reports of upregulation at early time points by mechanical wounding and herbivory (Glauser et al., 2009; Koo et al., 2009). Increases in bioactive JA (e.g., its isoleucine conjugate JA-Ile) cause degradation of JAZ1 by the SCF^coi1^—ubiquitin-proteasome pathway to de-repress the transcriptional activator MYC2 and activate early jasmonate response genes (Chung et al., 2009). The temporal dynamics in the caterpillar treatment resemble those in wounded tissue where high turnover of JAZ1 is reported within 1 h (Koo et al., 2009). The upregulation of JAZ1 by *P. syringae* is likely to reflect the negative feedback loop in which JA stimulates JAZ1 transcription.

Absence of JA metabolites in the caterpillar treatment at 48 h probably reflects the early induction and relaxation of jasmonate-related gene expression observed in this study and is consistent with the rapid turnover of JA, JA precursors, and JA conjugates reported by ourselves and others (Chung et al., 2009; Rehrig et al., 2014). The presence of both JA and SA metabolites in plants treated with *P. syringae* pv. tomato has been previously reported in response to the same pathogen (De Vos et al., 2005). The induction of SA and its glycoside reflects pathogen detection by the plant while induction of JA-related metabolites may reflect partial activation of the JA pathway by coronatine produced by the pathogen, consistent with the elevation of JAZ1 expression.

The *S. exigua* treatment was the only one to elicit transcriptional responses that clustered together with responses to jasmonates. This clustering also indicates that JA-responsive genes contributed significantly to this distinctiveness. Other genes in the array are involved in other JA-responses to stress (e.g., wounding, fungi, cold) but the close association in our study of the JA-responsive genes differentially expressed in response to *S. exigua* points to JA's prominent role in regulating the plant's response to this insect.

Caterpillar treatment induced expression of many genes involved in glucosinolate biosynthesis, whereas the pathogen largely reduced their expression. Glucosinolates are thioglycosides with defensive functions against insects and pathogens (Halkier and Gershenzon, 2006; Clay et al., 2009; Hopkins et al., 2009; Buxdorf et al., 2013). Arabidopsis leaves contain indolyl glucosinolates (IGs) with tryptophan-derived side chains and aliphatic glucosinolates (AGs) with methionine-derived side chains. When cells are damaged, glucosinolates are activated by myrosinases (beta-thioglucosidases) which cleave the glucose moiety and the remaining molecule quickly forms nitrile, thiocyanate, and isothiocyanate hydrolysis products whose formation is influenced by thiospecifiers in plants and insects. Although the caterpillar treatment induced the expression of many glucosinolate biosynthesis genes, glucosinolate metabolites were not statistically different than control values. This outcome may stem from a lack of metabolite induction due to the short term feeding in the caterpillar treatment (≤1 h feeding to achieve 10–30% leaf area removal) or an induction that occurred but had waned by the time of sampling. Pathogen treatment mostly reduced the expression of many glucosinolate biosynthesis genes. Levels of glucosinolates in these plants were also lower than in the caterpillar treatment, except for higher levels of the phytoalexin camalexin.

Plant responses to insects and biotrophic pathogens have been attributed to separate and antagonistic signaling pathways (JA vs. SA), but there are now many examples of the involvement of JA, SA, and ethylene signaling systems in plant responses to pathogens. For example, levels of both JA and SA are increased by *P. syringae* in Arabidopsis (Fan et al., 2009), and several pathovars of *P. syringae* produce the virulence factor coronatine that enhances JA signaling by targeting the physical interaction of JA-Ile and COI1 with JAZ1 to accelerate JAZ degradation and release MYC2, activating transcription of early JA response genes (Katsir et al., 2008; Melotto et al., 2008). As a result, resistance to some pathogens is lowered when function of the JA response pathway is compromised in Arabidopsis, e.g., in *jar1* mutants where JA cannot be converted to its active form JA-Ile, and in *coi1* mutants where JA-Ile/JAZ/COI1 interactions do not occur to cause JAZ degradation. In addition, the systemic signaling necessary for SAR appears to start as a JA signal that is transduced by auxin and IGs to increase SA in systemic tissues (Truman et al., 2010). Furthermore, ethylene, elicited by virulent pathogens and herbivores, mediates both JA and SA signaling (Groen et al., 2013).

### Effect of pre-treatment with caterpillars on susceptibility to avirulent and virulent *p. syringae*

Pre-treating Arabidopsis plants with caterpillar feeding before inoculation with avirulent or virulent *P. syringae* had significant effects on subsequent pathogen growth and the timing of expression of the *PR1* gene. Growth of avirulent *P. syringae* colonies was less on insect-treated plants by day 3pi. Expression of PR1 increased throughout the experiment on all plants inoculated with avirulent bacteria, but exceeded controls in insect-treated plants only on day 2pi. Hence decreased avirulent populations on day 3pi were associated with increased PR1 expression on day 2pi. Virulent *P. syringae* populations grew better than avirulent populations on both treated and untreated plants but leveled off on control plants and were growing significantly less well on insect-treated plants by day 3pi. The insect-treated plants exhibited significantly less PR1 expression on day 3pi as well. Hence insect treatment seems to have reduced the plant's response to virulent bacteria by day 3pi.

Causality for these patterns cannot be assigned to PR1 without manipulating its expression. Nonetheless, the fact that avirulent bacteria grew less well overall and elicited an earlier increase in PR1 expression than did virulent bacteria is in accord with expectations (Pieterse et al., 2012). Prior insect feeding appears to have augmented the earlier response to avirulent bacteria and may have contributed to lower avirulent colony growth. Prior feeding by caterpillars had no impact on either PR1 expression or colony growth in plants challenged with virulent bacteria until day 3pi, at which time increased colony growth was associated with reduced PR1 expression. Clearly prior insect feeding can influence performance of both bacterial strains, but the effects must involve the timing of the plants' response to the bacteria and probably different biochemical and genetic mechanisms, of which PR1 expression is only part of the story. It should be no surprise that the outcomes of complex interactions such as these, involving the intersection of many signaling systems, are context-dependent (Reymond et al., 2000; Aldea et al., 2005; Fan et al., 2009; Pieterse et al., 2012; Dinh et al., 2013; Groen et al., 2013; Rehrig et al., 2014).

In summary, the responses of Arabidopsis to caterpillar and pathogen attack may vary at the level of defense genes and chemical phenotype, but there is much broader overlap in signaling pathways and molecules than we realized. This overlap is especially true if we take into account the timing of their responses relative to attack. Many of the differences we see in plant responses to insects and biotrophic pathogens may largely be a matter of timing, i.e., when samples are collected after or during attack, rather than simply the identity of elicitors. The observation that multiple signaling pathways interact to produce unique phenotypes suggests that when specific responses develop, their regulation is complex and best understood by characterizing the expression of many genes and metabolites. The use of one or a few “marker” genes, or “typical” metabolites, may not provide enough useful information, especially when not conducted over a relevant time course.

Studies comparing plant responses to multiple stresses along with the response time course are critical to understanding how plants organize responses at the molecular through organismal levels to different stresses in agronomic and natural settings. Information obtained from these studies is necessary to efforts to improve resistance of crops, and our observation that insect attack can alter plant resistance to bacterial pathogens has the potential to compromise broad-based resistance to biotic stresses in the field. Constraints on plant responses to multiple stresses may also play an important role in shaping ecological communities.

## Author contributions

Heidi M. Appel: Helped design and conduct experiments, prepared manuscript, analyzed and interpreted data. Shahina B. Maqbool: Prepared material for and performed all microarray experiments, analyzed microarray data, wrote microarray portion of methods section, edited manuscript. Surabhi Raina: Prepared material for the microarray experiments, edited manuscript. Guru Jagadeeswaran: Prepared material for the microarray experiments, edited manuscript. Biswa R. Acharya: Prepared material for the microarray experiments, edited manuscript. John C. Hanley: Conducted HPLC-MS analysis of plant metabolites, assisted with analysis and interpretation of metabolite data. Kathryn P. Miller: Helped design and conduct caterpillar pretreatment experiment. Leonard Hearnes: Conducted statistical analysis of microarray and metabolite data, wrote statistics methods. A. Daniel Jones: Supervised HPLC-MS analysis of plant metabolites and interpretation of metabolite data, edited manuscript, co-authored funding for project. Ramesh Raina: Supervised microarray project, assisted with analysis and interpretation of the microarray data, edited manuscript, co-authored funding for project. Jack Schultz: Helped design experiments, assisted with the analysis and interpretation of data, conducted cluster analysis, edited manuscript, co-authored funding for project.

### Conflict of interest statement

The authors declare that the research was conducted in the absence of any commercial or financial relationships that could be construed as a potential conflict of interest.
